# Cuff meshoma post-laparoscopic sacrocolpopexy: vaginal-endoscopic mesh excision

**DOI:** 10.1007/s00192-023-05591-5

**Published:** 2023-07-06

**Authors:** Themistoklis Mikos, Iakovos Theodoulidis, George Kioussis, Tilemachos Karalis, Sofia Papaioannou, Grigoris F. Grimbizis

**Affiliations:** 1 1st Department of Obstetrics & Gynecology, Aristotle University of Thessaloniki, “Papageorgiou” General Hospital, Ring Road Neas Efkarpias, 56403 Thessaloniki, Greece; 2 Radiology Laboratory, Papageorgiou General Hospital, Thessaloniki, Greece

**Keywords:** Sacrocolpopexy, Mesh erosion, Mesh excision, Cuff meshoma, Vaginoscopy, Vaginal-endoscopic approach

## Abstract

**Introduction and hypothesis:**

The objective was to present endoscopic images of a meshoma and describe the complete excision of a complicated mesh after sacrocolpopexy (SCP) using a combined vaginal-endoscopic technique.

**Methods:**

We present a video documentation of an innovative technique. A 58-year-old woman was referred with painless, foul-smelling vaginal discharge and recurrent vaginal mesh erosions. She had undergone a laparoscopic SCP 12 years ago and her symptoms had begun 5 years ago. A pre-operative MRI scan revealed a cuff meshoma and an inflammatory sinus around the mesh extending from the cuff to the sacral promontory. Under general anesthesia, a 30° hysteroscope was inserted transvaginally into the sinus, where the retained mesh was seen in the form of a shrunken meshoma, and then the mesh arms were recognized extending cephalad into a sinus tract. Under direct endoscopic visualization, the mesh was carefully mobilized at its highest point with the use of laparoscopic grasping forceps. Then, the mesh was dissected with hysteroscopic scissors in close proximity to the bone. No peri-operative complications were recognized.

**Results:**

A combined vaginal-endoscopic approach was successfully used to remove an eroded mesh and cuff meshoma after SCP.

**Conclusion:**

This procedure offers a minimally invasive, low-morbidity, and rapid-recovery approach.

**Supplementary information:**

The online version contains supplementary material available at 10.1007/s00192-023-05591-5.

## Introduction

The prolapse of the vaginal vault has been defined as "the descent of the vaginal apex" and it entails the loss of apical vaginal support [1]. Although historically, the prevalence of vault prolapse ranges from 0.2% to 43.0% [2], more recent data estimate its incidence to be 11.6% after hysterectomy for prolapse and 1.8% for other pathological conditions [3]. One of the most common surgical procedures for treating vaginal vault prolapse after hysterectomy is sacrocolpopexy (SCP) with synthetic mesh, which is currently regarded as the gold standard with reported success rates ranging from 95 to 100% [4]. However, mesh SCP is related to unique postoperative complications such as mesh erosions into the vagina or neighboring organs occurring in 4.2% of the patients, or even more infrequent events such as sacral osteomyelitis [5–7]. Abdominal excision, transvaginal excision, and endoscopy-assisted transvaginal excision are three surgical methods that have been used to treat mesh erosion [8]. A meshoma is a clinical condition that was first reported by Amid to describe the situation where a mesh is folded and wrinkled into a process that continues until the mesh is wadded up into a ball [9]. Meshomas have been attributed with causing chronic pelvic pain after mesh repair of abdominal wall hernias [9]. To our knowledge a meshoma has never been reported after an SCP. The purpose of this study was to present endoscopic images of a meshoma post-SCP and to describe the complete excision of a complicated mesh after SCP using a combined vaginal-endoscopic technique.

## Materials and methods

A 58-year-old woman (gravida 2, para 2, BMI 18.8 kg/m^2^), presented to a tertiary referral center with 5-year history of chronic vaginal infections with foul-smelling vaginal discharge. She had undergone laparoscopic sacrocolpopexy 10 years prior to a referral for vaginal vault prolapse, following vaginal hysterectomy and native tissue anterior–posterior repair for stage III anterior wall and stage III uterine prolapse a year earlier. Numerous antibiotics had been used to treat her for 5 years without improvement, and she had a history of unsuccessful attempts at vaginal mesh removal (4 and 2 years before the current referral). A pelvic examination revealed a 0.5-cm sinus tract near the center of the vaginal cuff but no visible mesh erosion. Ultrasound examination and MRI showed the mesh above the vaginal cuff to be wadded up into a ball (meshoma), infection of the near tissues, and a sinus around the mesh extending up to the level of the sacral promontory. After extended counseling the patient opted for complete removal of the mesh (vaginal, endoscopic, laparoscopic or open, with full previous bowel preparation and cystoscopy with ureter indocyanine green (ICG) infusion/prophylactic stenting of the ureters), as her symptoms were seriously restricting her everyday activities.

Under general anesthesia, a 17F 70° cystoscope was used to exclude any bladder erosion, to ensure bilateral ureteral patency, and to infuse the right ureter with ICG. The vaginal cuff was well visualized and the centralized sinus tract orifice was easily identified. Then, the sinus tract was transvaginally marked with two Vicryl 1–0 sutures at 3 and 9 o’clock and dilated with Hegar dilators so that the 5-mm 12° hysteroscope could be easily passed. The endoscope was advanced into the tract in order to locate the mesh. The mesh was wadded up into a ball (meshoma) above the vaginal cuff, and infection covered most of the mesh and the surrounding tissues. Using forceps, outbound traction was applied, and the mesh was isolated from the surrounding tissues with hysteroscopic scissors. At this point, the meshoma had been freed apart from the surrounding tissues, and the ascending arms of the mesh could be seen following the trajectory of the sinus tract upward. Continuous endoscopic irrigation and repeated regrasping and pulling of the mesh provided further access to the mesh higher up in the sinus tract. At some point, the anchoring sutures to the periosteum of the promontory were recognized. At this level, the mesh was clear of debris, white in color, and the surrounding tissues appeared to be healthy and intersected within the pores and the sutures. As the limits of safe resection were reached, the mesh was transected using hysteroscopic scissors at the highest possible point (approximately 2 cm from the sacrum) and removed. No additional dissection was performed because the infected portion had been removed. The vaginal cuff was left open and allowed to heal by secondary intention. A 12F Foley was placed into the sinus as a surgical drain. Afterward, diagnostic laparoscopy and visualization of the ureters with RUBINA technology, cyanide blue test, and bubble test were performed. No intraoperative complications were recognized. The patient was discharged from the hospital after 3 days, with the bladder catheter removed on the 2nd postoperative day and the surgical drain removed on the 3rd.

## Results

The patient reported no further vaginal discharge at her 8-week postoperative follow-up. The vaginal cuff had completely healed, and the vault support remained adequate.

## Discussion

Mesh erosion is most likely caused by mesh inflammation and bacterial contamination [7]. Culligan et al. also noticed an increased risk of mesh erosion in patients undergoing concomitant hysterectomy at the time of sacrocolpopexy (27.3% vs 1.3%, *p* 0.001), supporting the idea that bacterial contamination from vaginal flora exposure contributes to mesh erosion [10]. Therefore, there are certain cases in which vaginal excision of the mesh erosion and re-epithelialization is not enough owing to pre-existent infection, and a more aggressive approach, including full mesh excision, may be appropriate for these patients.

It appears, however, that another significant parameter is the grade of integration of the mesh with the surrounding tissues. In our case the incorporation of the mesh never really took place. Thus, a sinus tract was created around the mesh immediately after the operation. Another consequence of non-integration of the mesh is the creation of the meshoma. As factors such as no fixation or insufficient fixation of the mesh, or inadequate dissection before mesh fixation to the vagina that predispose to meshoma creation are not usually encountered in SCP, a meshoma is rarely recognized after an SCP. In our case, however, the mesh was not only wrapped in a retroperitoneal fibrous tube but it was wadded into a ball exactly above the vaginal cuff, indicating that the distal sutures anchored to the vagina have either dissolved (the absorbable ones) or simply torn through the tissues (the non-absorbable ones), giving the non-integrated mesh the opportunity to fold.

Clinically, it is important to diagnose both the contamination of the mesh and the extent of its incorporation into the tissues. Recurrent mesh erosions are indicative of mesh contamination, whereas the use of CT/MRI may assist in the pre-operative diagnosis of sinus tract formation, a finding that suggests non-integration of the mesh. Moreover, the absence of chronic pain could be another factor indicating poor mesh integration.

## Conclusion

We present a meshoma post-sacrocolpopexy demonstrating recurrent painless vaginal mesh erosion and chronic foul-smelling vaginal discharge. This meshoma was developed in a non-integrated mesh surrounded by a sinus tract extending from the cuff to the promontory. A combined vaginal-endoscopic approach was successfully used to remove the meshoma and the full length of the mesh, offering the patient a minimally invasive, low-morbidity, and rapid-recovery approach.

### Supplementary Information

Below is the link to the electronic supplementary material.

Supplementary file1 (MP4 92024 KB)
